# Diagnostic accuracy of delirium diagnosis in pediatric intensive care: a systematic review

**DOI:** 10.1186/s13054-014-0489-x

**Published:** 2014-09-26

**Authors:** Alia Daoud, Jonathan P Duff, Ari R Joffe, for the Alberta Sepsis Network

**Affiliations:** Faculty of Medicine and Dentistry, University of Alberta, 8440 112 Street, Edmonton, AB T6G 2B7 Canada; Department of Pediatrics, Stollery Children’s Hospital and University of Alberta, 8440 112 Street, Edmonton, AB T6G 2B7 Canada; Department of Pediatrics, 4-546 Edmonton Clinic Health Academy, 11405 87 Avenue, Edmonton, AB T6G 1C9 Canada

## Abstract

**Introduction:**

Delirium is common in adult intensive care, with validated tools for measurement, known risk factors and adverse neurocognitive outcomes. We aimed to determine what is known about pediatric delirium in the pediatric intensive care unit (PICU).

**Methods:**

We conducted a systematic search for and review of studies of the accuracy of delirium diagnosis in children in the PICU. Secondary aims were to determine the prevalence, risk factors and outcomes associated with pediatric delirium. We created screening and data collection tools based on published recommendations.

**Results:**

After screening 145 titles and abstracts, followed by 35 full-text publications and reference lists of included publications, 9 reports of 5 studies were included. Each of the five included studies was on a single index test: (1) the Pediatric Anesthesia Emergence Delirium Scale (PAED; for ages 1 to 17 years), (2) the Pediatric Confusion Assessment Method for the Intensive Care Unit (p-CAM-ICU; for ages ≥5 years), (3) the Cornell Assessment of Pediatric Delirium (CAP-D; a modification of the PAED designed to detect hypoactive delirium), (4) the revised Cornell Assessment of Pediatric Delirium (CAP-D(R)) and (5) clinical suspicion. We found that all five studies had a high risk of bias on at least one domain in the Quality Assessment of Diagnostic Accuracy Studies-2 (QUADAS-2). Sample size, sensitivity, specificity, and effectiveness (correct classification divided by total tests done) were: PAED 144, 91%, 98%, <91% (>16% of scores required imputation for missing data); p-CAM 68, 78%, 98%, 96%; CAP-D 50, 91%, 100%, 89%; CAP-D (R) 111, and of assessments 94%, 79%, <82% (it is not clear if any assessments were not included); and clinical suspicion 877, N/A (only positive predictive value calculable, 66%). Prevalence of delirium was 17%, 13%, 28%, 21%, and 5% respectively. Only the clinical suspicion study researchers statistically determined any risk factors for delirium (pediatric risk of mortality, pediatric index of mortality, ventilation, age) or outcomes of delirium (length of stay and mortality).

**Conclusion:**

High-quality research to determine the accuracy of delirium screening tools in the PICU are required before prevalence, risk factors and outcomes can be determined and before a routine screening tool can be recommended. Direct comparisons of the p-CAM-ICU and CAP-D(R) should be performed.

**Electronic supplementary material:**

The online version of this article (doi:10.1186/s13054-014-0489-x) contains supplementary material, which is available to authorized users.

## Introduction

The American Psychiatric Association’s *Diagnostic and Statistical Manual of Mental Disorders, Fourth Edition* (DSM-IV), characterizes *delirium*, also known as *acute brain dysfunction*, as including four key features [1]. The definition includes (1) acute onset (usually hours to days) with a fluctuating course during the day, (2) a disturbance of consciousness, (3) change in cognition and (4) evidence of a causative medical condition, substance intoxication or medication side effect determined from the history, physical examination or laboratory tests. Delirium is common in adult intensive care, and there are validated tools for measurement as well as known risk factors and associated adverse neurocognitive outcomes [2,3]. A validated diagnostic method for delirium in the pediatric population has yet to become part of daily clinical practice. This is problematic, as the correct diagnosis of delirium in children enables early intervention to avoid the adverse effects of undiagnosed and untreated delirium. Moreover, the prevalence, risk factors, treatments and outcomes of pediatric delirium have not been determined. To study them, accurate, practicable screening tests are required.

To determine what is known about the diagnosis of delirium in the pediatric intensive care unit (PICU), we conducted a systematic review of the literature on the accuracy of diagnostic tests for delirium in children in the PICU. A secondary objective was to determine the risk factors for, outcomes of, and treatments for delirium in the PICU in the studies we identified for inclusion. We hypothesized that we would find validated and accurate diagnostic tests for delirium in the PICU. We identified only five accuracy studies, each of which had a high risk of bias and examined in isolation a different screening test for delirium. We make suggestions for future research needed in this field.

## Materials and methods

### Ethics review

We conducted a systematic review of diagnostic accuracy studies for delirium in the PICU. The University of Alberta Health Research Ethics Board waived the requirement for review because we used only publicly available information.

### Review question

We sought to determine the accuracy of the screening index test to diagnose delirium in children in the PICU as compared to a reference test (a “gold standard” test used for diagnosing delirium). To determine which studies to include, we used the explicit eligibility criteria described below.

1. The study had to be an accuracy study of delirium in which an index test for delirium was compared to a reference standard test used to diagnose delirium in the PICU. This type of study was searched for using any of the following keywords: accuracy, sensitivity, specificity, likelihood ratio, inter-rater,intra-rater, reliability, agreement and inter-observer, intra-observer [4]. The topic of delirium was screened for using the term delirium, and using synonyms, including acute brain dysfunction, withdrawal, acute encephalopathy and septic encephalopathy [2,3].
2. The patients studied had to be children, defined as neonates to 18 years old, and for whom data had to be reported separately from adult patients in the study (if applicable).
3. The study had to have been done with patients during their stay in the PICU.
4. The publications had to describe research studies; commentaries, reviews and editorials were excluded.
5. The studies had to have been published between January 1980 and May 2013. We later extended the PubMed search to June 2014.

Abstracts and titles were screened for these criteria by the three authors independently. The full texts of the potentially relevant abstracts and titles then were screened for these criteria by the three authors independently. The reference lists of the included publications were screened to identify additional publications for potential inclusion. The full texts of the additional publications thus identified were then reviewed, and, if potentially relevant, the decision whether to include them was made independently by the three authors. The final list of included publications was agreed upon by all authors.

### Literature search

The search strategy is shown in Additional file 1. Comprehensive search strategies were developed by an information specialist using a combination of subject headings and keywords and adapted for six electronic bibliographic databases. Searches were conducted in the following electronic databases: MEDLINE (via Ovid, 1946 to May Week 1 2013), Embase (via Ovid, 1980 to 2013 Week 19), PsycINFO (via Ovid, 1806 to May Week 1 2013), Health and Psychosocial Instruments (HaPI; via Ovid, 1985 to April 2013), CINAHL Plus with Full Text (via EBSCOhost, 1937 to May 2013) and PubMed (1980 to June 2014). No language or publication-type restrictions were applied. References were downloaded into EndNote X4.0.2 (Thomson Reuters, New York, NY, USA).

### Data collection

A data collection tool (available as Additional file 2) was created prior to the literature search, agreed upon by all three authors and included all elements suggested by the STARD statement (Statement for Reporting of Diagnostic Accuracy) [5], the Quality Assessment of Diagnostic Accuracy Studies (QUADAS-2) tool [6], the Cochrane Diagnostic Test Accuracy Working Group statement on systematic reviews of diagnostic test accuracy [4] and the Guidelines for Reporting Reliability and Agreement Studies [7]. The information collected included study population descriptions, participant recruitment and data collection methods, reference standards, details of index tests, biases present, results (including accuracy and reliability measures and patient flow) and study quality (risk of bias and applicability). Two authors (AD, ARJ) completed the data collection tool independently, and discrepancies were resolved by discussion and consensus. Our data collection tool, shown in Additional file 2, gives the details of our assessment method and definitions.

To assess quality, we used the QUADAS-2 tool. This tool asks signaling questions in each of four risk of bias domains and three applicability domains. For example, to assess risk of bias under the patient selection domain, questions include “Was a consecutive or random sample of patients enrolled?” and “Did the study avoid inappropriate exclusions?” The index test domain questions include “Were the index test results interpreted without knowledge of the results of the reference standard?” and “If a threshold was used, was it pre-specified?” To assess applicability, under the patient selection domain, the question asked is “Are there concerns that the included patients do not match the review question?” Under the reference standard domain, the question asked is “Are there concerns that the target condition as defined by the reference standard does not match the review question?” The QUADAS-2 tool is shown in Additional file 2.

### Statistics

We planned to determine the following for each index test.

1. We assessed the accuracy of index tests by analyzing sensitivity, specificity, positive and negative predictive values and likelihood ratios. We also looked at the impact of inconclusive tests on the index test’s accuracy [8].
2. Reliability and agreement (interrater and intrarater) of index tests were used as indicators of the amount of measurement error inherent in the index test score. κ-statistics for reliability were described with CIs. κ-values of 0.6 to 0.8 were considered sufficient for group-level comparisons; however, for individual diagnoses and important decisions, the κ-value should be at least 0.9.
3. We assessed the risk factors for, prevalence of (based on the reference standard), treatment of and outcome of delirium reported in the identified diagnostic accuracy studies using the individual study definitions and statistical tests employed therein.

Additional statistical analyses were planned if adequate data were found. These analyses are described in Additional file 3.

## Results

### Literature review

In the literature search, we identified 145 titles and abstracts for review. The three authors independently reviewed these and collectively identified 30 potentially relevant publications. The full texts of these were screened for eligibility, and seven were identified for inclusion. Screening of the references in these seven studies led us to review another five full-text articles, among which two were identified for inclusion. Overall, nine reports were included once deemed to meet the predefined eligibility criteria. In these nine articles, data from five studies are reported. In each of these five studies, researchers examined the diagnostic accuracy of an individual index test for PICU delirium [9-17].

### Study descriptions

The methods used in the five studies are described in Table 1 [9-12,17]. In each study, researchers examined a unique index test, making direct comparisons of index tests and collection of summary statistics impossible (see Additional file 3 for analyses that were planned). All age groups were included, except for the Pediatric Confusion Assessment Method for the Intensive Care Unit (p-CAM-ICU [10]; limited to patients developmentally age ≥5 years) and the Pediatric Anesthesia Emergence Delirium Scale (PAED [9,16]; studied in children ages 1 to 17 years). A proportion of eligible patients were excluded from analysis because of missing data in two of the studies (20% for p-CAM-ICU, 11% for the Cornell Assessment of Pediatric Delirium (CAP-D) [11]). In the PAED study, many patients had missing data that were imputed (6% excluded had missing data, and another >16% had imputed missing data). Four studies were prospective, and one was retrospective (data from 20% of patients “reconstructed” retrospectively for PAED score). The methodological quality of each study according to QUADAS-2 criteria is shown in Table 2. Each study had high risk of bias in at least one domain. Only the p-CAM-ICU raised a high level of concern regarding applicability (due to only 6% of patients being ventilated and children under a developmental age of 5 years being excluded). The specific biases present are detailed in Additional file 3.

**Table 1:** **Study descriptions** ^**a**^

**Publication**	**Setting**	**Index tests**		**Reference tests**		**Dates of study**	**Eligibility**	**Recruitment; data acquisition method**	**Final number of patients (eligible but excluded** ***n*** **, ** **%)**
		**Name**	**No. of raters (training)**	**Name**	**No. of raters (training)**				
Janssen *et al*., 2011 [9,16]	University hospital	PAED	Unclear (“interns”)	DSM-IV and multidisciplinary meeting	1 (psychiatrist, with input from PICU team)	November 2006 through February 2010	All PICU patients ages 1 to 17 yr without deep sedation; if elective surgery, PICU stay >48 hr	Consecutive; retrospective	144 (*n* =10, 6% where PAED could not be rated)
Smith *et al*., 2011 [10]	University hospital	p-CAM-ICU	6 (4 bedside nurses, 2 intensivists)	DSM-IV	2 (psychiatrists)	1 July 2008 through 30 March 2009	All PICU patients ages ≥5 yr	Consecutive (except weekends and holidays); prospective	68 (*n* =17, 20%; no paired assessment =13, assessments >3 hr apart =3, withdrawn =1)
Silver *et al*., 2012 [11]	University hospital	CAP-D	2 (1 intensivist, 1 resident)	DSM-IV	4 (2 psychiatrists, 2 fellows)	Unspecified 6-week period	All PICU patients with RASS score above −4	Consecutive; prospective	50 (*n* =6, 11% with incomplete data)
Traube *et al*., 2013 [17]	University hospital	CAP-D(R)	>100 (bedside nurses)	DSM-IV	6 (psychiatrists)	March 2012 through May 2012	All PICU patients with RASS score above −4	Consecutive; prospective	111 (0)
Schieveld *et al*., 2007 [12-15]	University hospital	Clinical suspicion^b^	Unclear (intensivists)	DSM-IV and multidisciplinary meeting	1 (psychiatrist, with input from PICU team)	January 2002 through December 2005	All PICU patients	Consecutive; prospective	877 (0)

^a^CAP-D, Cornell Assessment of Pediatric Delirium (a modification of the PAED designed to detect hypoactive delirium); CAP-D(R), Cornell Assessment of Pediatric Delirium, Revised (modifications include changes in the original response options to better capture a fluctuating course, addition of a question to detect alteration in cognitive functioning, and training materials that include lists of “anchor points” describing age-appropriate developmental expectations); DSM-IV, *Diagnostic and Statistical Manual of Mental Disorders, Fourth Edition* [1]; PAED, Pediatric Anesthesia Emergence Delirium Scale; p-CAM-ICU, Pediatric Confusion Assessment Method for the Intensive Care Unit; PICU, Pediatric intensive care unit; RASS: Richmond Agitation–Sedation Scale. ^b^Clinical suspicion was defined as “confusion, agitation, moaning, discomfort, or behavioral disturbances with no acceptable medical explanation; or, failure of standard analgosedative treatment”.

**Table 2:** **Methodological quality of the included studies** ^**a**^

**Study index test**	**Study flow diagram (Y/N)**	**QUADAS-2 quality criteria: risk of bias** ^**b**^				**QUADAS-2 quality criteria: applicability concern** ^**b**^		
		**Patient selection**	**Index test**	**Reference standard**	**Flow and timing**	**Patient selection**	**Index test**	**Reference standard**
PAED [9,16]	N	High	Unclear	Low	Unclear	Low	Low	Low
p-CAM-ICU [10]	Y	Low	Low	Low	High	High	Low	Low
CAP-D [11]	N	Unclear	Low	Unclear	High	Low	Low	Low
CAP-D(R) [17]	Y	Low	High	Low	Unclear	Low	Low	Low
Clinical suspicion [12-15]	N	Low	High	Unclear	High	Low	Low	Low

^a^CAP-D, Cornell Assessment of Pediatric Delirium; CAP-D(R), Cornell Assessment of Pediatric Delirium, Revised; PAED, Pediatric Anesthesia Emergence Delirium Scale; p-CAM-ICU, Pediatric Confusion Assessment Method for the Intensive Care Unit; QUADAS-2, Quality Assessment of Diagnostic Accuracy Studies-2; Y/N, Yes or no. ^b^A description of the QUADAS-2 criteria for risk of bias and applicability concerns [6] is given in the data collection tool (available as Additional file 2). A low risk of bias indicates better quality compared to high or unclear risk of bias. A low applicability concern indicates better quality compared to high or unclear applicability concern*.*

### Study descriptive results

The descriptive results of the included studies are shown in Table 3. The admission diagnoses of patients in two of the studies may not have been representative of most PICUs: asthma was the admitting diagnosis for 12% of patients in the p-CAM-ICU study [10], and oncology patients accounted for 26% of the sample in the CAP-D study [11]. In most of the studies, the researchers did not report patient comorbidities, risk factors for delirium, treatment of delirium or outcomes. Neurologic comorbidity was reported in only the CAP-D study [11] and the revised CAP-D study (CAP-D(R) [17]), with developmental delays present in 12 patients (24%) and 22 patients (20%), respectively.

**Table 3:** **Descriptive results of the included studies** ^**a**^

**Index test**	**Age range**	**Comorbidities**	**Admission diagnoses (top four)**	**Risk factors (including comorbidities)**	**Outcomes identified**	**Treatment described**	**Prevalence of delirium**			
							**Overall**	**Hyperactive**	**Hypoactive**	**Mixed**
PAED [9,16]	1 to 17 yr	No data	Respiratory (32%), neurological (23%), circulatory (17%), surgical (8%)	Not analyzed	Not analyzed	No data	26/154 (17%)	18/154 (12%)	4/154 (3%)	4/154 (3%)
p-CAM-ICU [10]	≥5 yr developmentally	No data	Congenital heart disease surgery (18%), asthma (12%), traumatic brain injury (9%), septic shock (9%)	No data	No data	No data	9/68 (13%)	–	–	–
CAP-D [11]	3 mo to 21 yr	Developmental delay in 12 (24%)	Oncology (26%), cardiac (16%), neurosurgical (16%), infectious (10%)	No data	No data	No data	14/50 (28%)	2/50 (4%)	6/50 (12%)	6/50 (12%)
CAP-D(R) [17]	0 to 21 yr	Developmental delay in 22 (20%)	Postoperative (50%), respiratory insufficiency (45%), infectious/inflammatory (34%), neurosurgical (27%)	High PIM II, age <13 yr, developmental delay, respiratory support (no statistical comparison made)	No data	No data	51/248 assessments (20.6%)	–	–	–
Clinical suspicion [12-15]	3 mo to 17 yr	No data	Respiratory (30%), neurologic (40%), circulatory (20%), surgical (7.5%)	High PIM, PRISM, age, ventilation, diagnostic category (neurologic)	Higher mortality, more PICU days	Haloperidol (2/28 with dystonic reactions), risperidone (*n* =11)	40/877 (5%)	14/877 (2%)	9/877 (1%)	17/877 (2%)

^a^CAP-D, Cornell Assessment of Pediatric Delirium; CAP-D(R), Cornell Assessment of Pediatric Delirium, Revised; PAED, Pediatric Anesthesia Emergence Delirium Scale; p-CAM-ICU, Pediatric Confusion Assessment Method for the Intensive Care Unit; PICU, Pediatric intensive care unit; PIM, Pediatric Index of Mortality; PRISM, Pediatric Risk of Mortality.

Statistical data for risk factors, outcomes and treatment of delirium were reported in only one study (clinical suspicion) [12-15]. In that study, risk factors identified were high Pediatric Index of Mortality score (median 5.8 vs 1.6, *P* <0.001), high Pediatric Risk of Mortality score (PRISM II; median 11.3 vs 2.6, *P* <0.001), relatively older age (7.7 (SD 5.8) years vs 4.3 (SD 4.8) years, *P* <0.001), mechanical ventilation (34 (85%) vs 333 (39.8%), *P* <0.0005) and admission diagnosis more with a neurological disorder 16 patients (40%) vs 159 (19%), less with a respiratory disorder 12 (30%) vs 434 (52%); *P* =0.018) [13]. In follow-up of that study extending for another 14 months and limited to patients 1 to 18 years of age, the researchers reported on patients with delirium (*n* =49) compared to a random PICU control group (*n* =98). In the follow-up report, admission diagnosis and PRISM II were no longer found to be risk factors [14]. Delirium was associated with a longer PICU stay (median 11 days vs 3 days, *P* <0.001) and higher mortality (5 (12.5%) vs 36 (4.3%), *P* =0.016) [13]. Again, the follow-up report of that study confirmed an independent association of delirium with a longer PICU stay (+2.39 days); however, the mortality difference was not confirmed (2.04% with delirium vs 6.12% without delirium) [14]. The treatment used for delirium was haloperidol or risperidone. Among the 27 patients given haloperidol, 2 (7%) of had acute dystonia as a side effect. The prevalence of delirium varied between studies from 5% to 28% and included hyperactive, hypoactive and mixed subtypes. The low prevalence of 5% was likely due to the study design, in which only patients suspected of having delirium by attending intensivists were referred for the gold standard test [12].

### Index test performance

The performance of each index test is shown in Table 4. The PAED, CAP-D and CAP-D(R) had high sensitivity, and the PAED, CAP-D and p-CAM-ICU had high specificity; however, there is overlap of the 95% CIs of these point estimates. The sensitivity and specificity of the “clinical suspicion” study could not be calculated. The sensitivity and specificity for PAED and CAP-D may have been inflated because of the valid inconclusive results in the studies, with index test yield (that is, the percentage of patients who had the index test who were included in the sensitivity and specificity calculations) and effectiveness (index test correct classification/total index tests done) of about 90%. Valid inconclusive results are those where the index or reference test is neither clearly positive nor clearly negative (that is, an intermediate result, and the result is excluded from the study after enrollment). The CAP-D(R) sensitivity and specificity could not be calculated based on the number of patients, as only data on the number of total assessments (248 assessments in 111 patients) were provided, and whether 248 represented all the assessments was not stated. In addition, the PAED was assessed in the study of the CAP-D, with reported sensitivity of 50% (7 of 14) and specificity of 100% (36 of 36), indicating that the sensitivity may have been overestimated in the main (retrospective) PAED study [11]. Only the p-CAM-ICU and CAP-D(R) studies determined interrater reliability between two raters, using the κ-statistic, with results of 0.96 (95% CI 0.74 to 1.0) and 0.94 (no 95% CI provided), respectively, indicating excellent interrater reliability. No measure of index test agreement was assessed in any of the studies.

**Table 4:** **Performance of the index tests** ^**a**^

**Study index test**	**Cross-tabulation reported**	**Sensitivity,** ***n/N*** **, % ** **(95% ** **CI)**	**Specificity,** ***n/N*** **, % ** **(95% ** **CI)**	**PPV,** ***n/N*** **, ** **% (95% ** **CI)**	**NPV,** ***n/N*** **, % ** **(95% ** **CI)**	**Positive likelihood ratio**	**Negative likelihood ratio**	**Valid inconclusive results** ^**b**^	
								**Yield**	**Effectiveness**
PAED^c^ [9,16]	No	21/23, 91% (72% to 99%)	119/121, 98% (94% to 99.9%)	21/23, 91% (72% to 99%)	119/121, 98% (94% to 99.9%)	53.7	0.09	<144/154, <93.5%	<140/154, <91%
p-CAM-ICU [10]	No	7/9, 78% (40% to 97%)	58/59, 98% (91% to 99.9%)	7/8, 88% (51% to 99.9%)	58/60, 97% (88% to 99.8%)	46	0.22	68/68, 100%	65/68, 96%
CAP-D^d^ [11]	No	21/23, 91% (72% to 99%)	54/54, 100% (94% to 100%)	21/21, 100% (86% to 100%)	54/56, 96% (87% to 99.7%)	91	0.09	50/56, 89%	50/56, 89%
CAP-D(R) [17]	No	48/51 assessments, 94.1% (84% to 99%)	156/197 assessments, 79.2% (74% to 85%)	48/89 assessments, 54% (44% to 64%)	156/159 assessments, 98% (94% to 99.6%)	4.5	0.07	248/?, Unclear	<204/248 assessments, <82%
Clinical suspicion [12-15]	No	N/A	N/A	40/61, 66% (53% to 76%)	N/A	N/A	N/A	N/A	N/A

^a^CAP-D, Cornell Assessment of Pediatric Delirium; CAP-D(R), Cornell Assessment of Pediatric Delirium, Revised; N/A, Data not collected and thus could not be calculated; NPV, Negative predictive value; PAED, Pediatric Anesthesia Emergence Delirium Scale; p-CAM-ICU, Pediatric Confusion Assessment Method for the Intensive Care Unit; PPV, Positive predictive value. ^b^Valid inconclusive results are those where the index or reference test is neither clearly positive nor clearly negative (that is, an intermediate result, and the result is excluded from the study after enrollment). Yield is the percentage of patients who had the index test who are included in the sensitivity and specificity calculations; Effectiveness is index test correct classification divided by total index tests done [8]. The PAED scores have a “<” sign because imputed values due to missing data were used for up to 16% of each item in the PAED score. The CAP-D(R) values have a “<” sign because whether all assessments were included in the study was not stated. ^c^We did not consider this study sufficiently powered to evaluate the Delirium Rating Scale (DRS) 88 or the DRS-Revised, because there was too much missing data. The yields were 103/154 (67%) and 73/154 (47%), respectively, even before considering imputed values due to missing data used for >50% of some items in these scores. It is important to note that the performance of the PAED was not as good in the study by Silver *et al*. [11]: sensitivity =7/14 (50%) (95% CI 27% to 73%), specificity =36/36 (100%) (95% CI 92% to 100%), PPV =7/7 (100%) (95% CI 68% to 100%), NPV =36/43 (84%) (95% CI =70% to 92%), positive likelihood ratio =50, negative likelihood ratio =0.5. ^d^Only the p-CAM-ICU and CAP-D(R) determined interrater reliability between two raters using the κ-statistic: 0.96 (95% CI 0.74 to 1.0) in 146 paired assessments and 0.94 (no 95% CI reported) in 70 paired assessments, respectively. Only the CAP-D(R) determined the interrater reliability of the gold standard: κ =0.96 (95% CI 0.79 to 1.00) in 38 paired psychiatric evaluations.

## Discussion

Delirium in adult intensive care is common (incidence estimates range from 45% to 87%), and there are well-validated screening tools for diagnosis. It has known risk factors and is associated with increased intensive care and hospital lengths of stay, increased mortality, and long-term cognitive impairment [2,3]. In our systematic review, we found only five studies of the diagnostic accuracy of tests for delirium in the PICU [9-12,17]. In these five studies, delirium was common, with a prevalence of 13% to 28% (excluding the study of “clinical suspicion,” in which partial and differential verification bias limited sensitivity for delirium), including hyperactive, hypoactive and mixed subtypes. In general, the studies were small and single-centered and had methodological weaknesses and high risk of bias. In each study, researchers examined a different index test, and, other than the “clinical suspicion” index test study, the researchers did not examine risk factors, treatments or outcomes of delirium. The sensitivity and specificity of the PAED (retrospectively in patients ≥1 year of age), the CAP-D (prospectively in all age groups) and the p-CAM-ICU (prospectively in patients developmentally ages ≥5 years) were high; however, these data should be interpreted cautiously, given the study limitations, including high risk of bias and small numbers, and, in addition, one group could not confirm high sensitivity for the PAED [11]. There were no direct comparisons of the index tests, except in one study in which both the CAP-D and PAED were compared. In that study, the CAP-D had much better accuracy. Interrater reliability was examined only for the p-CAM-ICU and CAP-D(R), and the reliability was excellent.

We are aware of two other systematic reviews of delirium in children, neither of which critically examined studies of the diagnostic accuracy of tests for delirium in the PICU. Neither of these two reviews referenced any studies that we did not include [18,19]. In one, the authors searched for publications about pediatric delirium (not limited to the PICU) published between 1980 and 2009 and found only small case series and case reports. They concluded, “Delirium is an important but neglected disorder of childhood associated with significant morbidity and high mortality. Current clinical practice for management is based on slim empirical evidence” ([18], p 337). In the other systematic review, the authors focused on delirium in the PICU and searched the literature published between March 2009 and March 2011 (that is, following the first systematic review dates). In that review, the authors found only two observational studies, one of which examined p-CAM-ICU and was included in our present review [10] and one of which was the follow-up study to the “clinical suspicion” study included in our present review [14]. The authors of the latter review concluded that “there are still important, yet unresolved issues, regarding pathophysiology and biomarkers, risk factors, early detection, and appropriate treatment” ([19], p 1106). We are also aware of two narrative reviews of delirium in the PICU [20,21]. Neither of those reviews references any study not included in our present review, nor is either of them focused on the diagnostic accuracy of delirium screening tools in the PICU.

The importance of delirium in adult intensive care is clear. Delirium is both common and commonly overlooked, and it has adverse consequences, including prolonged ventilation, increased hospital and ICU lengths of stay, increased health care costs, long-term cognitive impairment and physical disability. The duration of delirium is independently associated with mortality [2,3,22]. The main screening tools for adults include the CAM-ICU (pooled sensitivity 76% to 80%, pooled specificity 96%) and the Intensive Care Delirium Screening Checklist (ICDSC; pooled sensitivity 74% to 80%, pooled specificity 75% to 82%), which are used to assess delirium over the course of 1 minute (CAM-ICU) and 8 to 24 hours (ICDSC), respectively [3]. The CAM-ICU requires interaction with the patient, but the ICDSC does not (observation only) [3].

Similarly, in our present review, we found that delirium in the PICU is common and that it likely has adverse consequences, including increased hospital and PICU lengths of stay and possibly increased mortality. The main screening tools either require (p-CAM-ICU) or do not require (PAED, CAP-D, CAP-D(R)) patient interaction. Both the p-CAM-ICU and the CAP-D(R) are quick screening tools (taking less than 2 minutes to complete) [17,21], have high sensitivity (78% and 94%, respectively) and high specificity (91% and 79%, respectively) and have excellent interrater reliability. However, methodological risk of bias and the limited number of single-center studies preclude making conclusions about their true performance characteristics. The weaknesses of the PAED include its low sensitivity for hypoactive delirium, variable sensitivity in the two studies (91% and 50%) [9,11] and the major methodological biases of the favorable study (retrospective methodology and imputed data for >16% of patients).

There are limitations to this systematic review. The quality of information in systematic reviews is only as good as the included studies. The quality of our five included studies was only modest, with small patient numbers and high risk of bias. The studies are the first in the field and likely reflect the difficulty of performing costly, time-consuming clinical research in critical care. Nevertheless, the results of our systematic review suggest that investment in high-quality comparative and confirmatory studies are needed. The small studies we identified did not allow for examination of accuracy in different subgroups of patients based on age, diagnosis and so forth. The literature search methods for diagnostic accuracy studies are not well worked out. We followed recommendations in the literature, but it is possible that we missed important studies. We did not screen for descriptive studies of delirium in the PICU not focused on accuracy. Thus, It is possible that there are data about prevalence, risk factors, treatment and outcomes that we did not find in our literature search. The strengths of our study include its systematic search strategy, the use of published recommendations for assessing accuracy studies and their quality in systematic reviews, and the use of prespecified study screening and data collection tools employed independently by three and two authors, respectively (to minimize the risk of subjective bias in study selection and quality assessment). Our conclusions are therefore based on a transparent methodology using evidence-based guidelines for systematic assessment of the available literature.

## Conclusions

High-quality research to determine the accuracy of delirium screening tools in the PICU are required before prevalence, risk factors, treatment and outcomes can be determined and before routine screening can be recommended. Nevertheless, use of a screening tool to detect delirium in the PICU should be a priority of future research, given the likely high prevalence and adverse consequences of the diagnosis. In particular, direct comparisons of the most promising tools, the p-CAM-ICU and CAP-D(R), should be performed. Future research should also be carried out to determine whether prevention and/or treatment strategies for delirium can change the outcomes of PICU patients.

## Key messages

- Delirium is common in pediatric intensive care, with a prevalence of 13% to 28%.
- High-quality research to determine the accuracy of delirium screening tools in the PICU are required before prevalence, risk factors, treatments and outcomes can be determined.
- There are two promising screening tools for pediatric delirium in intensive care that warrant further study: p-CAM-ICU and CAP-D(R).

## Additional files

Additional file 1:
**The search strategy used in the systematic review.** This file provides details of the search strategy. Additional file 2:
**The data collection tool used to abstract study data in the systematic review.** This file provides the tool used to abstract all data from the included publications in the review. Additional file 3:
**Supplemental statistical analysis and results section of the systematic review.** This file provides details of planned analyses that were not performed due to lack of data in the included publications in the review, and details of the specific biases present in each included study.

## References

[1] American Psychiatric Association (2000). Diagnostic and Statistical Manual of Mental Disorders.

[2] Cavallazzi R, Saad M, Marik PE (2012). Delirium in the ICU: an overview. Ann Intensive Care.

[3] Brummel NE, Vasilevskis EE, Han JH, Boehm L, Pun BT, Ely EW (2013). Implementing delirium screening in the ICU: secrets to success. Crit Care Med.

[4] Leeflang MMG, Deeks JJ, Gatsonis C, Bossuyt PMM, on behalf of the Cochrane Diagnostic Test Accuracy Working Group (2008). Systematic reviews of diagnostic test accuracy. Ann Intern Med.

[5] Bossuyt PM, Reitsma JB, Bruns DE, Gatsonis CA, Glasziou PP, Irwig LM, Moher D, Rennie D, de Vet HCW, Lijmer JG (2003). The STARD statement for reporting studies of diagnostic accuracy: explanation and elaboration. Ann Intern Med.

[6] Whiting PF, Rutjes AWS, Westwood ME, Mallett S, Deeks JJ, Reitsma JB, Leeflang MMG, Sterne JAC, Bossuyt PMM, and the QUADAS-2 Group (2011). QUADAS-2: a revised tool for the quality assessment of diagnostic accuracy studies. Ann Intern Med.

[7] Kottner J, Audigé L, Brorson S, Donner A, Gajewski BJ, Hróbjartsson A, Roberts C, Shoukri M, Streiner DL (2011). Guidelines for Reporting Reliability and Agreement Studies (GRRAS) were proposed. J Clin Epidemiol.

[8] Shinkins B, Thompson M, Mallett S, Perera R (2013). Diagnostic accuracy studies: how to report and analyse inconclusive test results. BMJ.

[9] Janssen NJJF, Tan EYL, Staal M, Janssen EPCJ, Leroy PLJM, Lousberg R, van Os J, Schieveld JNM (2011). On the utility of diagnostic instruments for pediatric delirium in critical illness: an evaluation of the Pediatric Anesthesia Emergence Delirium Scale, the Delirium Rating Scale 88, and the Delirium Rating Scale-Revised R-98. Intensive Care Med.

[10] Smith HAB, Boyd J, Fuchs C, Melvin K, Berry P, Shintani A, Eden SK, Terrell MK, Boswell T, Wolfram K, Sopfe J, Barr FE, Pandharipande PP, Ely EW (2011). Diagnosing delirium in critically ill children: validity and reliability of the Pediatric Confusion Assessment Method for the Intensive Care Unit. Crit Care Med.

[11] Silver G, Traube C, Kearney J, Kelly D, Yoon MJ, Moyal WN, Gangopadhyay M, Shao H, Ward MJ (2012). Detecting pediatric delirium: development of a rapid observational assessment tool. Intensive Care Med.

[12] Schieveld JNM, Leroy PLJM, van Os J, Nicolai J, Vos GD, Leentjens AFG (2007). Pediatric delirium in critical illness: phenomenology, clinical correlates and treatment response in 40 cases in the pediatric intensive care unit. Intensive Care Med.

[13] Schieveld JNM, Lousberg R, Berghmans E, Smeets I, Leroy PLJM, Vos GD, Nicolai J, Leentjens AFG, van Os J (2008). Pediatric illness severity measures predict delirium in a pediatric intensive care unit. Crit Care Med.

[14] Smeets IAP, Tan EYL, Vossen HGM, Leroy PLJM, Lousberg RHB, van Os J, Schieveld JNM (2010). Prolonged stay at the paediatric intensive care unit associated with paediatric delirium. Eur Child Adolesc Psychiatry.

[15] Leentjens AFG, Schieveld JNM, Leonard M, Lousberg R, Verhey FRJ, Meagher DJ (2008). A comparison of the phenomenology of pediatric, adult, and geriatric delirium. J Psychosom Res.

[16] Blankespoor RJ, Janssen NJJF, Wolters AMH, van Os J, Schieveld JNM (2012). Post-hoc revision of the Pediatric Anesthesia Emergence Delirium rating scale: clinical improvement of a bedside tool?. Minerva Anestesiol.

[17] Traube C, Silver G, Kearney J, Patel A, Atkinson TM, Yoon MJ, Halpert S, Augenstein J, Sickles LE, Li C, Greenwald B (2014). Cornell Assessment of Pediatric Delirium: a valid, rapid, observational tool for screening delirium in the PICU. Crit Care Med.

[18] Hatherill S, Flisher AJ (2010). Delirium in children and adolescents: a systematic review of the literature. J Psychosom Res.

[19] Creten C, van der Zwaan S, Blankespoor RJ, Leroy PLJM, Schieveld JNM (2011). Pediatric delirium in the pediatric intensive care unit: a systematic review and an update on key issues and research questions. Minerva Anestesiol.

[20] Smith HAB, Fuchs DC, Pandharipande PP, Barr FE, Ely EW (2011). Delirium: an emerging frontier in the management of critically ill children. Anesthesiol Clin.

[21] Smith HAB, Brink E, Fuchs DC, Ely EW, Padharipande PP (2013). Pediatric delirium: monitoring and management in the pediatric intensive care unit. Pediatr Clin North Am.

[22] Reade MC, Finfer S (2014). Sedation and delirium in the intensive care unit. N Engl J Med.

