# Supplementary material for: Diagnostic accuracy of delirium diagnosis in pediatric intensive care: a systematic review
Source: Crit Care. 2014 Sep 26;18(5):489. doi: 10.1186/s13054-014-0489-x (PMC4207322; doi:10.1186/s13054-014-0489-x)
Supplement: Additional file 1: — The search strategy used in the systematic review. This file provides details of the search strategy. [file 13054_2014_489_MOESM1_ESM.pdf]

DIAGNOSTIC ACCURACY OF DELIRIUM DIAGNOSIS IN PEDIATRIC INTENSIVE CARE: A SYSTEMATIC REVIEW

ESM_1_Joffe:
The search strategy used in the systematic review.

Authors: Alia Daoud BSc[1], Jonathan P Duff MD[1,2], Ari R Joffe MD[1,2]

Affiliations: 1. University of Alberta, Faculty of Medicine and Dentistry; 2. University of Alberta, Department of Pediatrics and Stollery Children's Hospital.

Corresponding Author: Ari R Joffe MD; 4-546 Edmonton Clinic Health Academy; 11405 87 Avenue; Edmonton, Alberta, Canada; T6G 1C9. Phone: 780 2485435. Email: ari.joffe@albertahealthservices.ca Fax: 780 4073214.

PICU Delirium Searches

Topic: Delirium (all causes) in PICU (not NICU or neonatal); include adolescents
Databases: MEDLINE, EMBASE, CINAHL, PsycINFO, and HaPI
Limits: 1980-current
Study design/publication type: all (including letters, notes, commentary)

PubMed will be searched for last 180 days.

Search Summary

Database	Results	Duplicates between databases removed (de-duped)
**MEDLINE**	51	51
**EMBASE**	63	34
**PyscINFO**	40	40
**HaPI**	0	0
**CINAHL**	3	2
**PubMed**	55	8
**TOTALS**	212	135

Notes for all searches:

- Limits: date range: 1980-current
- No language or publication type limits – PsycINFO search brought in many book references
- Scoping searches showed limiting to PICU rather than an 18 and under population was too narrow, therefore age (infant [not neonate], child, adolescent) was combined with delirium. Using PICU, references to adolescents were not retrieved.
- With poor indexing in this topic area (diagnostic accuracy), PubMed was run from 1980-current instead of the originally planned last 180 days as a check and balance to the other database retrievals.

Search Methodology

Comprehensive search strategies were developed by an information specialist (TD) using a combination of subject headings and keywords and adapted for 6 electronic bibliographic databases. Searches were conducted in the following electronic databases: MEDLINE (Ovid, 1946 to May Week 1 2013), EMBASE (Ovid, 1980 to 2013 Week 19), PsycINFO (Ovid, 1806 to May Week 1 2013), Health and Psychosocial Instruments HaPI (Ovid, 1985 to April 2013), CINAHL Plus with Full Text (EBSCOhost) (1937 to May 2013), and PubMed. No language or publication type restrictions were applied. References were downloaded into EndNote 4X.0.2 (Thomson Reuters).

Search Strategies

Ovid MEDLINE 1946 to May Week 1 2013
OvidSP version: Version: OvidSP_UI03.08.01.105, SourceID 57688
Search date: 10.05.2013
Limits: 1980-current
Results: 51 De-duped: 51

PICU delirium1

1. Delirium/
2. deliri$.tw.
3. deleri$.tw.
4. Confusion/
5. (acute adj2 confusion$).tw.
6. (acute adj2 brain syndrome).tw.
7. (acute adj2 organic psychosyndrome).tw.
8. (acute adj2 psycho-organic syndrome).tw.
9. (acute adj2 brain failure).tw.
10. (terminal$ adj restless$).tw.
11. toxic confus$.tw.
12. or/1-11
13. exp child/
14. infant/
15. (child or children or infant? or baby or babies).tw.
16. Adolescent/
17. adolescen$.tw.
18. young adult?.tw.
19. teen$.tw.
20. youth?.tw.
21. or/13-20
22. Psychological tests/
23. exp Neuropsychological Tests/
24. neuropsychologic$ test?.tw.
25. ((screen$ or evaluat$ or assess$ or diagnos$ or rating or rate or measur$ or case finding) adj5 (index or test$ or instrument$ or inventor$ or battery or batteries or tool? or scale? or checklist? or check list? or schedule?)).tw.
26. (Paediatric Confusion Assessment Method or Pediatric Confusion Assessment Method or pCAM-ICU).tw.
27. (Cornell Assessment of Pediatric Delirium or CAP-D).tw.
28. (Pediatric Anesthesia Emergence Delirium or PAED).tw.
29. (Delirium Rating Scale or DRS-88).tw.
30. (Delirium Rating Scale-Revised or DRS-R-98 or Delirium Rating Scale-Revised Version-1998).tw.
31. (Delirium Diagnostic Tool-Provisional or DDT-Pro).tw.
32. Inferential Confusion Scale.tw.
33. (Nursing Delirium Screening Scale or Nu-DESC).tw.
34. Confusion Assessment Method.tw.
35. Cognitive Test for Delirium.tw.
36. or/22-35
37. exp "Sensitivity and Specificity"/
38. sensitivity.tw.
39. specificity.tw.
40. ((pre-test or pretest) adj probability).tw.
41. post-test probability.tw.
42. likelihood ratio$.tw.
43. (reliability or reproducib$ or inter rate or interrater or valid$ or test retest or predictive or psychometric$).mp.
44. or/37-43
45. and/12,21,36,44
46. limit 45 to yr="1980 -Current"

47. remove duplicates from 46

EMBASE 1980 to 2013 Week 19
OvidSP version: Version: OvidSP_UI03.08.01.105, SourceID 57688
Search date: 10.05.2013
Limits: 1980-current
Results: 63 De-duped: 34

PICU delirium2
1. Delirium/
2. Postoperative delirium/
3. deliri$.tw.
4. deleri$.tw.
5. exp Confusion/
6. (acute adj2 confusion$).tw.
7. (acute adj2 brain syndrome).tw.
8. (acute adj2 organic psychosyndrome).tw.
9. (acute adj2 psycho-organic syndrome).tw.
10. (acute adj2 brain failure).tw.
11. (terminal$ adj restless$).tw.
12. toxic confus$.tw.
13. or/1-12
14. exp child/
15. (child or children or infant? or baby or babies).tw.
16. exp adolescent/
17. adolescen$.tw.
18. young adult?.tw.
19. teen$.tw.
20. youth?.tw.
21. or/14-20
22. exp psychologic test/
23. neurologic examination/
24. neuropsychological test/
25. neuropsychologic$ test?.tw.
26. ((screen$ or evaluat$ or assess$ or diagnos$ or rating or rate or measur$ or case finding) adj5 (index or test$ or instrument$ or inventor$ or battery or batteries or tool? or scale? or checklist? or check list? or schedule?)).tw.
27. (Paediatric Confusion Assessment Method or Pediatric Confusion Assessment Method or pCAM-ICU).tw.
28. (Cornell Assessment of Pediatric Delirium or CAP-D).tw.
29. (Pediatric Anesthesia Emergence Delirium or PAED).tw.
30. (Delirium Rating Scale or DRS-88).tw.
31. (Delirium Rating Scale-Revised or DRS-R-98 or Delirium Rating Scale-Revised Version-1998).tw.
32. (Delirium Diagnostic Tool-Provisional or DDT-Pro).tw.
33. Inferential Confusion Scale.tw.
34. (Nursing Delirium Screening Scale or Nu-DESC).tw.
35. Confusion Assessment Method.tw.
36. Cognitive Test for Delirium.tw.
37. or/22-36
38. exp "Sensitivity and Specificity"/

39. sensitivity.tw.
40. specificity.tw.
41. ((pre-test or pretest) adj probability).tw.
42. post-test probability.tw.
43. likelihood ratio$.tw.
44. *Diagnostic Accuracy/
45. (reliability or reproducib$ or inter rate or interrater or valid$ or test retest or predictive or psychometric$).mp.
46. or/38-45
47. and/13,21,37,46
48. limit 47 to yr="1980 -Current"
49. limit 48 to exclude medline journals
50. 48 not 49
51. remove duplicates from 50

PsycINFO 1806 to May Week 1 2013
OvidSP version: Version: OvidSP_UI03.08.01.105, SourceID 57688
Search date: 10.05.2013
Limits: 1980-current
Results: 40 De-duped: 40
Note: no diagnostic evaluation filter used. Use of filter gave 3 results removing documents that may have relevance.

PICU delirium3
1. Delirium/
2. deliri$.tw.
3. deleri$.tw.
4. (acute adj2 confusion$).tw.
5. (acute adj2 brain syndrome).tw.
6. (acute adj2 organic psychosyndrome).tw.
7. (acute adj2 psycho-organic syndrome).tw.
8. (acute adj2 brain failure).tw.
9. (terminal$ adj restless$).tw.
10. toxic confus$.tw.
11. or/1-10
12. (child or children or infant? or baby or babies).tw.
13. adolescen$.tw.
14. young adult?.tw.
15. teen$.tw.
16. youth?.tw.
17. or/12-16
18. psychological assessment/
19. exp neuropsychological assessment/
20. cognitive assessment/
21. neuropsychologic$ test?.tw.
22. ((screen$ or evaluat$ or assess$ or diagnos$ or rating or rate or measur$ or case finding) adj5 (index or test$ or instrument$ or inventor$ or battery or batteries or tool? or scale? or checklist? or check list? or schedule?)).tw.
23. (Paediatric Confusion Assessment Method or Pediatric Confusion Assessment Method or pCAM-ICU).tw.

24. (Cornell Assessment of Pediatric Delirium or CAP-D).tw.
25. (Pediatric Anesthesia Emergence Delirium or PAED).tw.
26. (Delirium Rating Scale or DRS-88).tw.
27. (Delirium Rating Scale-Revised or DRS-R-98 or Delirium Rating Scale-Revised Version-1998).tw.
28. (Delirium Diagnostic Tool-Provisional or DDT-Pro).tw.
29. Inferential Confusion Scale.tw.
30. (Nursing Delirium Screening Scale or Nu-DESC).tw.
31. Confusion Assessment Method.tw.
32. Cognitive Test for Delirium.tw.
33. or/18-32
34. and/11,17,33
35. limit 34 to yr="1980 -Current"
36. remove duplicates from 35

Health and Psychosocial Instruments (HaPI) 1985 to April 2013
OvidSP version: Version: OvidSP_UI03.08.01.105, SourceID 57688
Search date: 10.05.2013
Limits: 1980-current
Results: 0 De-duped: 0
Note: this is a database of tests; used to find additional tests

PICU delirium4
1. deliri$.mp.
2. deleri$.tw.
3. (acute adj2 confusion$).tw.
4. (acute adj2 brain syndrome).tw.
5. (acute adj2 organic psychosyndrome).tw.
6. (acute adj2 psycho-organic syndrome).tw.
7. (acute adj2 brain failure).tw.
8. (terminal$ adj restless$).tw.
9. toxic confus$.tw.
10. or/1-9
11. (child or children or infant? or baby or babies).tw.
12. adolescen$.tw.
13. young adult?.tw.
14. teen$.tw.
15. youth?.tw.
16. or/11-15
17. psychological test?.tw.
18. neuropsychologic$ test?.tw.
19. ((screen$ or evaluat$ or assess$ or diagnos$ or rating or rate or measur$ or case finding) adj5 (index or test$ or instrument$ or inventor$ or battery or batteries or tool? or scale? or checklist? or check list? or schedule?)).tw.
20. (Paediatric Confusion Assessment Method or Pediatric Confusion Assessment Method or pCAM-ICU).tw.
21. (Cornell Assessment of Pediatric Delirium or CAP-D).tw.
22. (Pediatric Anesthesia Emergence Delirium or PAED).tw.
23. (Delirium Rating Scale or DRS-88).tw.
24. (Delirium Rating Scale-Revised or DRS-R-98 or Delirium Rating Scale-Revised Version-1998).tw.
25. (Delirium Diagnostic Tool-Provisional or DDT-Pro).tw.
26. Inferential Confusion Scale.tw.

27. (Nursing Delirium Screening Scale or Nu-DESC).tw.
28. Confusion Assessment Method.tw.
29. Cognitive Test for Delirium.tw.
30. or/17-29
31. and/10, 16, 30
32. limit 32 to yr="1980 -Current"

CINAHL Plus with Full Text
Interface: EBSCOhost, advanced search
Searched: 10.05.2013
Limits: 1980-current
Results: 3 De-duped: 2

#	Query	Limiters/Expanders	Results
S18	S5 AND S8 AND S13 AND S17	Limiters - Published Date from: 19800101-20131231; Exclude MEDLINE records Search modes - Boolean/Phrase	3
S17	S14 OR S15 OR S16	Search modes - Boolean/Phrase	230,219
S16	(MH "Instrument Validation")	Search modes - Boolean/Phrase	19,202
S15	(sensitivity or specificity) OR (pre-test w1 probability or pretest w1 probability or post-test w1 probability or likelihood w1 ratio) OR (reliability or reproducib* or inter rate or interrater or valid* or test retest or predictive or psychometric*)	Search modes - Boolean/Phrase	230,219
S14	(MH "Sensitivity and Specificity")	Search modes - Boolean/Phrase	40,385
S13	S9 OR S10 OR S11 OR S12	Search modes - Boolean/Phrase	150,046
S12	(Paediatric Confusion Assessment Method or Pediatric Confusion Assessment Method or pCAM-ICU) OR (Cornell Assessment of Pediatric Delirium or CAP-) OR (Pediatric Anesthesia Emergence Delirium or PAED) OR (Delirium Rating Scale or DRS-88) OR (Delirium Rating Scale-Revised or DRS-R-98 or Delirium Rating Scale-Revised Version-1998) OR (Delirium Diagnostic Tool-Provisional or DDT-Pro) OR Inferential Confusion Scale OR (Nursing Delirium Screening Scale or Nu-DESC) OR Confusion Assessment Method OR Cognitive Test for Delirium	Search modes - Boolean/Phrase	2,206
S11	(screen* w5 index or evaluat* w5 index or assess* w5 index or diagnos* w5 index or rating w5 index or rate w5 index or	Search modes - Boolean/Phrase	133,178

	measur* w5 index or case finding w5 index) OR (screen* w5 test* or evaluat* w5 test* or assess* w5 test* or diagnos* w5 test* or rating w5 test* or rate w5 test* or measur* w5 test* or case finding w5 test*) OR (screen* w5 instrument* or evaluat* w5 instrument* or assess* w5 instrument* or diagnos* w5 instrument* or rating w5 instrument* or rate w5 instrument* or measur* w5 instrument* or case finding w5 instrument*) OR (screen* w5 inventor* or evaluat* w5 inventor* or assess* w5 inventor* or diagnos* w5 inventor* or rating w5 inventor* or rate w5 inventor* or measur* w5 inventor* or case finding w5 inventor*) OR (screen* w5 battery or evaluat* w5 battery or assess* w5 battery or diagnos* w5 battery or rating w5 battery or rate w5 battery or measur* w5 battery or case finding w5 battery) OR (screen* w5 batteries or evaluat* w5 batteries or assess* w5 batteries or diagnos* w5 batteries or rating w5 batteries or rate w5 batteries or measur* w5 batteries or case finding w5 batteries) OR (screen* w5 tool* or evaluat* w5 tool* or assess* w5 tool* or diagnos* w5 tool* or rating w5 tool* or rate w5 tool* or measur* w5 tool* or case finding w5 tool*) OR (screen* w5 scale* or evaluat* w5 scale* or assess* w5 scale* or diagnos* w5 scale* or rating w5 scale* or rate w5 scale* or measur* w5 scale* or case finding w5 scale*) OR (screen* w5 checklist* or evaluat* w5 checklist* or assess* w5 checklist* or diagnos* w5 checklist* or rating w5 checklist* or rate w5 checklist* or measur* w5 checklist* or case finding w5 checklist*) OR (screen* w5 check list* or evaluat* w5 check list* or assess* w5 check list* or diagnos* w5 check list* or rating w5 check list* or rate w5 check list* or measur* w5 check list* or case finding w5 check list*) OR (screen* w5 schedule* or evaluat* w5 schedule* or assess* w5 schedule* or diagnos* w5 schedule* or rating w5 schedule* or rate w5 schedule* or measur* w5 schedule* or case finding w5 schedule*)		
S10	neuropsychologic* test*	Search modes - Boolean/Phrase	20,451
S9	(MH "Neuropsychological Tests")	Search modes - Boolean/Phrase	20,013
S8	S6 OR S7	Search modes - Boolean/Phrase	620,409
S7	child or children or infant? or baby or babies or adolescen* or young adult* or teen* or youth*	Search modes - Boolean/Phrase	578,526
S6	(MH "Child") OR (MH "Infant") OR (MH "Infant, Newborn") OR (MH "Adolescence+")	Search modes - Boolean/Phrase	495,697
S5	S1 OR S2 OR S3 OR S4	Search modes - Boolean/Phrase	5,219
S4	acute w2 confusion* or acute w2 brain syndrome or acute	Search modes -	275

	w2 organic psychosyndrome or acute w2 psycho-organic syndrome or acute w2 brain failure or terminal* w restless*	Boolean/Phrase	
S3	(MH "Confusion+")	Search modes - Boolean/Phrase	4,008
S2	deliri* or deleri*	Search modes - Boolean/Phrase	3,998
S1	(MH "Delirium")	Search modes - Boolean/Phrase	2,837

PubMed http://www.ncbi.nlm.nih.gov/pubmed/
Searched: 12.05.2013
Limits: 1980-current
Results: 55 De-duped: 8

Search	Add to builder	Query	Items found
#76	Add	Search (((#47) AND #50) AND #69) AND #73 Filters: Publication date from 1980/01/01 to 2013/12/31	55
#74	Add	Search (((#47) AND #50) AND #69) AND #73	56
#73	Add	Search (#71) OR #72	1452032
#72	Add	Search sensitivity[tiab] OR specificity[tiab] OR predictive value* OR likelihood ratio* OR pre-test probability[tiab] OR pretest probability[tiab] OR post-test probability[tiab] OR reliability[tiab] OR reproducib*[tiab] OR "inter rate*"[tiab] OR interrater[tiab] OR valid*[tiab] or test retest[tiab] OR predictive[tiab] or psychometric[tiab]	1318015
#71	Add	Search "Sensitivity and Specificity"[Mesh]	378819
#69	Add	Search ((((((((((((#33) OR #52) OR #53) OR #57) OR #58) OR #59) OR #61) OR #62) OR #63) OR #64) OR #65) OR #66) OR #67) OR #68	1652686
#68	Add	Search "Cognitive Test for Delirium"[tiab]	0
#67	Add	Search "Confusion Assessment Method"[tiab]	458
#66	Add	Search "Nursing Delirium Screening Scale"[tiab] OR Nu-DESC[tiab]	16
#65	Add	Search "Inferential Confusion Scale"[tiab]	0
#64	Add	Search "Delirium Diagnostic Tool-Provisional"[tiab] OR DDT-Pro[tiab]	1
#63	Add	Search "Delirium Rating Scale-Revised"[tiab] OR DRS-R-98 OR "Delirium Rating Scale-Revised Version-1998"[tiab]	61
#62	Add	Search "Delirium Rating Scale[tiab] OR DRS-88[tiab]	151
#61	Add	Search "Pediatric Anesthesia Emergence Delirium"[tiab] OR PAED[tiab]	71
#59	Add	Search "Cornell Assessment of Pediatric Delirium"[tiab] OR CAP-D[tiab]	12
#58	Add	Search "Paediatric Confusion Assessment Method"[tiab] OR "Pediatric Confusion Assessment Method"[tiab] or pCAM-ICU[tiab]	5
#57	Add	Search (#56) AND #55	1589185
#55	Add	Search index[tiab] OR test*[tiab] OR instrument*[tiab] OR inventor[tiab]* OR battery[tiab] OR batteries[tiab] OR tool*[tiab] OR scale[tiab]* OR	2638179

Search	Add to builder	Query	Items found
		checklist[tiab]* OR check list*[tiab] OR schedule*[tiab]	
#56	Add	Search screen*[tiab] OR evaluat*[tiab] OR assess*[tiab] OR diagnos*[tiab] OR rating[tiab] OR rate[tiab] OR measur*[tiab] OR case finding[tiab]	6607953
#53	Add	Search neuropsychologic* test*[tw]	23897
#52	Add	Search "Neuropsychological Tests"[Mesh]	62289
#33	Add	Search "Psychological Tests"[Mesh:NoExp]	32660
#50	Add	Search ((((#14) OR #18) OR #48) OR #27) OR #49	2832991
#49	Add	Search adolescen*[tiab] OR young adult*[tiab] OR teen*[tiab] OR youth*[tiab]	238034
#27	Add	Search "Adolescent"[Mesh]	1514909
#48	Add	Search child[tiab] or children[tiab] or infant*[tiab] or baby[tiab] or babies[tiab]	1068978
#18	Add	Search "Infant"[Mesh:NoExp]	608401
#14	Add	Search "Child"[Mesh]	1470006
#47	Add	Search (((#8) OR #42) OR #45) OR #46	12844
#46	Add	Search acute confusion*[tiab] OR acute brain syndrome[tiab] OR acute organic psychosyndrome[tiab] OR acute psycho-organic syndrome[tiab] OR acute brain failure[tiab] OR terminal* restless*[tiab] OR toxic confus*[tiab]	109
#45	Add	Search "Confusion"[Mesh:NoExp]	3489
#42	Add	Search deliri*[tiab] OR deleri*[tiab]	8302
#8	Add	Search delirium[mh]	5161

Search Summary:

Database	Date Searched	Number Retrieved	After Duplicate Removal
PubMed	11 June 2014	65	N/A
**Total**		**65**	

Database: PubMed

Search Title: Joffe-Diagnosing-delirium_UpdateJune2014_RF

Date Searched: June 11, 2014

Results: 65 [Note: 10 studies with a publication date year of 2013 or 2014]

Search	Add to builder	Query	Items found	Time
#34	Add	Search **#7 AND #13 AND #30 AND #33**	65	12:02:54
#33	Add	Search **#31 OR #32**	1578431	11:38:26
#32	Add	Search **sensitivity[tiab] OR specificity[tiab] OR predictive value* OR likelihood ratio* OR pre-test probability[tiab] OR pretest probability[tiab] OR post-test probability[tiab] OR reliability[tiab] OR reproducib*[tiab] OR "inter rate*"[tiab] OR interrater[tiab] OR valid*[tiab] or test retest[tiab] OR predictive[tiab] or psychometric[tiab]**	1435411	11:38:05
#31	Add	Search **"Sensitivity and Specificity"[Mesh]**	408808	11:37:50
#30	Add	Search **#14 OR #29 OR #15 OR #18 OR #19 OR #20 OR #21 OR #22 OR #23 OR #24 OR #25 OR #26 OR #27 OR #28**	1948678	11:37:32
#29	Add	Search **"Neuropsychological Tests"[Mesh]**	67541	11:33:40
#28	Add	Search **"Cognitive Test for Delirium"[tiab]**	0	11:31:46
#27	Add	Search **"Confusion Assessment Method"[tiab]**	544	11:31:39
#26	Add	Search **"Nursing Delirium Screening Scale"[tiab] OR Nu-DESC[tiab]**	22	11:31:25
#25	Add	Search **"Inferential Confusion Scale"[tiab]**	0	11:31:13
#24	Add	Search **"Delirium Diagnostic Tool-Provisional"[tiab] OR DDT-Pro[tiab]**	1	11:30:59
#23	Add	Search **"Delirium Rating Scale-Revised"[tiab] OR DRS-R-98 OR "Delirium Rating Scale-Revised Version-1998"[tiab]**	83	11:30:45
#22	Add	Search **"Delirium Rating Scale"[tiab] OR DRS-88[tiab]**	177	11:30:28
#21	Add	Search **"Pediatric Anesthesia Emergence Delirium"[tiab] OR PAED[tiab]**	85	11:29:34
#20	Add	Search **"Cornell Assessment of Pediatric Delirium"[tiab] OR CAP-D[tiab]**	15	11:29:21
#19	Add	Search **"Paediatric Confusion Assessment Method"[tiab] OR "Pediatric Confusion Assessment Method"[tiab] or pCAM-ICU[tiab]**	5	11:29:06
#18	Add	Search **#16 AND #17**	1883402	11:28:49
#17	Add	Search **index[tiab] OR test*[tiab] OR instrument*[tiab] OR inventor[tiab]* OR battery[tiab] OR batteries[tiab] OR tool*[tiab] OR**	3139202	11:27:47

Search	Add to builder	Query	Items found	Time
		**scale[tiab]* OR checklist[tiab]* OR check list*[tiab] OR schedule*[tiab]**		
#16	Add	Search **screen*[tiab] OR evaluat*[tiab] OR assess*[tiab] OR diagnos*[tiab] OR rating[tiab] OR rate[tiab] OR measur*[tiab] OR case finding[tiab]**	7129063	11:27:27
#15	Add	Search **neuropsychologic* test*[tw]**	30637	11:26:42
#14	Add	Search **"Psychological Tests"[Mesh:NoExp]**	33283	11:26:32
#13	Add	Search **#8 OR #9 OR #10 OR #11 OR #12**	2967725	11:26:21
#12	Add	Search **adolescen*[tiab] OR young adult*[tiab] OR teen*[tiab] OR youth*[tiab]**	260825	11:25:20
#11	Add	Search **"Adolescent"[Mesh]**	1582247	11:25:11
#10	Add	Search **child[tiab] or children[tiab] or infant*[tiab] or baby[tiab] or babies[tiab]**	1133397	11:24:59
#9	Add	Search **"Infant"[Mesh:NoExp]**	628844	11:24:49
#8	Add	Search **"Child"[Mesh]**	1522598	11:24:39
#7	Add	Search **#3 OR #4 OR #5 OR #6**	13929	11:22:17
#6	Add	Search **acute confusion*[tiab] OR acute brain syndrome[tiab] OR acute organic psychosyndrome[tiab] OR acute psycho-organic syndrome[tiab] OR acute brain failure[tiab] OR terminal* restless*[tiab] OR toxic confus*[tiab]**	113	11:21:56
#5	Add	Search **"Confusion"[Mesh:NoExp]**	3643	11:21:39
#4	Add	Search **deliri*[tiab] OR deleri*[tiab]**	9185	11:21:28
#3	Add	Search **delirium[mh]**	5593	11:21:13
