# Supplementary material for: Diagnostic accuracy of delirium diagnosis in pediatric intensive care: a systematic review
Source: Crit Care. 2014 Sep 26;18(5):489. doi: 10.1186/s13054-014-0489-x (PMC4207322; doi:10.1186/s13054-014-0489-x)
Supplement: Additional file 2: — The data collection tool used to abstract study data in the systematic review. This file provides the tool used to abstract all data from the included publications in the review. [file 13054_2014_489_MOESM2_ESM.pdf]

DIAGNOSTIC ACCURACY OF DELIRIUM DIAGNOSIS IN PEDIATRIC INTENSIVE CARE: A SYSTEMATIC REVIEW

ESM_2_Joffe:
The data collection tool used to abstract study data in the systematic review.

Authors: Alia Daoud BSc[1], Jonathan P Duff MD[1,2], Ari R Joffe MD[1,2]

Affiliations: 1. University of Alberta, Faculty of Medicine and Dentistry; 2. University of Alberta, Department of Pediatrics and Stollery Children's Hospital.

Corresponding Author: Ari R Joffe MD; 4-546 Edmonton Clinic Health Academy; 11405 87 Avenue; Edmonton, Alberta, Canada; T6G 1C9. Phone: 780 2485435. Email: ari.joffe@albertahealthservices.ca Fax: 780 4073214.

DATA COLLECTION TOOL

Title	
Authors	
Reference [Journal, Year, Volume: page numbers]	
**METHODOLOGY**	
Study Research Question	
Study setting	University hospital<br>Community hospital
Index Test Used	Name: ____________<br>Description in enough detail to replicate: Y/N<br>Categories (delirium/not delirium thresholds) defined: apriori/post-hoc<br>What was the threshold for delirium: ____________<br>Training of raters: Y/N/not stated<br>Number of raters: ______<br>Qualifications of raters: Research physician Staff intensivist Fellow Resident Bedside nurse Research nurse Parent Others:________<br>Cost per single index test: $________ Not mentioned
Study population: Inclusion (Indications for the Index Test)	Age: ________<br>Diagnoses: ____________<br>Co-morbidities: ____________
Study population: Exclusions (Indications for the Index Test)	Age: ________<br>Diagnoses: ____________<br>Co-morbidities: ____________
Date of Study	Start date: ____________<br>End date: ____________
Name/Type of Reference Test Used:	Name (eg. DSM criteria): ____________<br>Training of raters: Y/N/not stated<br>Qualifications of raters: Psychiatrist Fellow Resident Nurse Other:__________
Recruitment process	All PICU patients Referral for assessment<br>Other:
Patient Sampling Method	Consecutive<br>Non-consecutive (sub-selection): random/non-random<br>Please describe if non-random: ____________
Data Collection Process	Prospective/Retrospective

<table>
<tr><td colspan="3"><u>RESULTS</u></td></tr>
<tr><td rowspan="6">Patient Flow:</td><td>Flow diagram in paper</td><td>Y/N</td></tr>
<tr><td>Number eligible</td><td>n= ___________</td></tr>
<tr><td>Number meeting pre-specified exclusion criteria</td><td>n= ___________</td></tr>
<tr><td>Number enrolled (intention to diagnose)</td><td>n= ___________</td></tr>
<tr><td>Number of participants excluded from the study after enrollment:</td><td>n= ___________</td></tr>
<tr><td>Number of participants in final numbers for outcome (sensitivity/ specificity)</td><td>n= ___________</td></tr>
<tr><td>Reason for exclusion from the study after enrollment (and n)</td><td colspan="2">Invalid Inconclusive Result of Index Test:
a) Uninterpretable diagnostic feature:
-clinical feature hampers interpretation of test Y/N
n= ___________
Describe: ___________
-Obstructed test (interrupted) Y/N
n= ___________
Describe: ___________
-Questionable validity (an inadequate test procedure was conducted; not conducted to an acceptable standard) Y/N
n= ___________
Describe: ___________
b) Missing (not done): Y/N
n= ___________</td></tr>
<tr><td></td><td colspan="2">Valid Inconclusive Index Test (neither clearly positive nor negative; intermediate):
a) Continuous inconclusive test results (overlap): Y/N
n= ___________
Describe: ___________
b) Categorical inconclusive test results: Y/N
n= ___________
Describe: ___________</td></tr>
<tr><td></td><td colspan="2">Valid Inconclusive Reference Test (neither clearly positive nor negative):
a) Continuous inconclusive test results (overlap): Y/N
n= ___________
Describe: ___________
b) Categorical inconclusive test results: Y/N
n= ___________
Describe: ___________</td></tr>
<tr><td>Enrolled Participant Descriptions</td><td colspan="2"></td></tr>
</table>

Ages	
Illness(s)/condition(s) of participants enrolled	Admission diagnoses (top 4):<br><br>Co-morbidities (top 4):
Risk Factors for delirium:	**Factor Delirium No Delirium Risk**<br>Age<br>Male<br>Severity of illness<br>Diagnostic Category<br>Sepsis/fever/infection<br>Cardiac surgical<br>Neurosurgical<br>General Surgical<br>Trauma<br>TBI<br>Neurologic Medical<br>Respiratory<br>Other<br>Medications<br>Inotropes/Pressors<br>Benzodiazepines<br>Opiates<br>Dexmedetomidine<br>Other<br>Other conditions<br>Ventilated<br>Shock<br>ARDS<br>HBP<br>Electrolyte abn<br>Other:
Predisposing (pre-existing) co-morbidities of participants enrolled	**Factor Delirium No Delirium Risk**<br>Cognitive impairment<br>Seizure disorder<br>Stroke<br>Vision impaired<br>Hearing impaired<br>Other
The Outcomes of delirium:	**Outcome Delirium No Delirium Risk**<br>Mortality<br>PICU<br>Hospital<br>Other<br>LOS<br>Ventilation d<br>PICU d

	**Outcome Delirium No Delirium Risk**<br>Hospital d<br>Neurocognitive<br>PTSD<br>Other
Treatment of delirium described	Benzodiazepines:<br>Dexmedetomidine:<br>Haloperidol:<br>Atypical Antipsychotic:<br>Other:
The Incidence of any delirium (%)<br>(#patients with delirium diagnosis by reference test)/ (#patients)	(#pts with delirium by reference test)/(#pts enrolled in the study)= ________________<br>(#pts with delirium by reference test)/(#pts included in the study)= ________________
The Incidence of hyperactive delirium (%)	(#pts with delirium by reference test)/(#pts enrolled in the study)= ________________<br>(#pts with delirium by reference test)/(#pts included in the study)= ________________
The Incidence of hypoactive delirium (%)	(#pts with delirium by reference test)/(#pts enrolled in the study)= ________________<br>(#pts with delirium by reference test)/(#pts included in the study)= ________________
The Incidence of mixed delirium (%)	(#pts with delirium by reference test)/(#pts enrolled in the study)= ________________<br>(#pts with delirium by reference test)/(#pts included in the study)= ________________
Time Interval between repeated measurements	Of Index Test (Hours): Intra-rater:____________ Inter-rater:____________<br>Of Reference Test (Hours): Intra-rater:____________ Inter-rater:____________
Time Interval between the Index and Reference Test	< 12hr 12 to 24 hours >24 hours<br>Was there any treatment for delirium in between: Y/N

The presence of Types of BIAS

Spectrum Bias	Included patients do not represent the intended spectrum of severity for the target condition or alternative conditions (eg more advanced stages).	YES NO<br>Describe:
Selection Bias	Eligible patients are not enrolled consecutively or randomly	YES NO<br>Describe:
Index Test Information Bias	The index test results are interpreted with knowledge of the reference test results, or with more clinical information than in practice.	YES NO<br>Describe:
Misclassification Bias	The reference standard does not correctly classify patients with the target condition.	YES NO<br>Describe:

Context Bias	The prevalence of delirium is much different than expected in the PICU population	YES NO<br>Describe:
Partial Verification Bias	A non random set of patients does not undergo the reference standard.	YES NO<br>Describe:
Differential Verification Bias	A set of patients is verified with a different reference standard than another set, especially when this selection depends on the index test result (eg. index test influences decision to order reference test)	YES NO<br>Describe:
Incorporation Bias	The index test is incorporated in a composite reference standard	YES NO<br>Describe:
Disease Progression Bias	The patients' condition changes between administering the index test and the reference standard (i.e. when the period of time between the index and reference tests is too long; defined as >24hr apart, not on same day)	YES NO<br>Describe:
Reference Test Information Bias	The reference standard is interpreted knowing the index test results.	YES NO<br>Describe:
Excluded Data	Occurs when uninterpretable or intermediate test results and withdrawals are not included in the analysis.	YES NO<br>Describe:
Limited Challenge Bias	Patients with a specific condition known to adversely affect the way the index test works are excluded (eg. difficult to diagnose patients excluded).	YES NO<br>Describe:

Statistics: count each patient only once i.e. if 1 patient examined more than once, use first exam(iner) only, or report separate for each exam(iner) if available. If only median (IQR) (range) reported among examiners, then cannot create the cross-tabulation table, but can report the reported statistics by median (IQR) (range).

Fill the next page out for

a) **each index test used (if more than one was used in the study), and**
b) **each subgroup reported separately (eg. Individual raters; type of delirium [hyperactive, hypoactive, mixed]; type of participant (eg. Sepsis, Trauma, etc); center (if multicenter study)**
c) **any sensitivity analysis reported (eg. Including valid inconclusive results considered with positive or negative index test)**

This table and results are for: overall study / subgroup [describe: __________]/ sensitivity analysis [describe: ________________________________].

		REFERENCE TEST		Totals
		**+ for Delirium**	**- for Delirium**	
**INDEX TEST (Name: __________)**	**+ for Delirium**	a=	b=	
	**- for Delirium**	c=	d=	
	**Inconclusive (Uninterpretable)**			
	**Totals**			e

Was this cross-tabulation reported in the paper?	Yes No
Sensitivity (The proportion of patients with positive results on the reference test that are also positive on the index test)= a/(a+c)	Mentioned in paper:_________<br>95% CI in paper: _______ or no<br>Calculated from cross tabulation: ___
Specificity (The proportion of patients with negative results on the reference test that are also negative on the index test) = d/(b+d)	Mentioned in paper:_________<br>95% CI in paper: _______ or no<br>Calculated from cross tabulation: ___
Positive Predictive Value = a/(a+b)	Mentioned in paper:_________<br>95% CI in paper: _______ or no<br>Calculated from cross tabulation: ___
Negative Predictive Value = d/(c+d)	Mentioned in paper:_________<br>95% CI in paper: _______ or no<br>Calculated from cross tabulation: ___
Positive Likelihood ratio = sensitivity / (1 - specificity). If a cell is 0, use 0.5 added to each cell in table to calculate LR.	Mentioned in paper:_________<br>95% CI in paper: _______ or no<br>Calculated from cross tabulation: ___
Negative Likelihood ratio = (1 – sensitivity) / specificity. If a cell is 0, use 0.5 added to each cell in table to calculate LR.	Mentioned in paper:_________<br>95% CI in paper: _______ or no<br>Calculated from cross tabulation: ___
If there are valid inconclusive results: what was the index test yield = % of patients included in the sensitivity and specificity calculations.	__________%
If there are valid inconclusive results: what was the index test effectiveness = correct classification/total tests done = (a+d)/e.	__________%
If there are valid inconclusive results: what was the sensitivity and specificity if the results are grouped with positive index test results.	Sensitivity: _______<br>Specificity: _______
If there are valid inconclusive results: what was the sensitivity and specificity if the results are grouped with negative index test results.	Sensitivity: _______<br>Specificity: _______

QUADAS-2 Quality Criteria

**Patient Selection [3 signaling question, 1 risk of bias question, 1 applicability question]**			
Was a consecutive or random sample of patients enrolled?	YES	NO	UNCLEAR
Was a case-control design avoided?	YES	NO	UNCLEAR
Did the study avoid inappropriate exclusions?	YES	NO	UNCLEAR
Could the selection of patients have introduced bias?	High	Low	UNCLEAR
Are there concerns that the included patients do not match the review question?	High	Low	UNCLEAR
**Index Test [2 signaling question, 1 risk of bias question, 1 applicability question]**			
Were the index test results interpreted without knowledge of the results of the reference standard?	YES	NO	UNCLEAR
If a threshold was used, was it pre-specified?	YES	NO	UNCLEAR
Could the conduct or interpretation of the index test have introduced bias?	High	Low	UNCLEAR
Are there concerns that the index test, its conduct, or interpretation differ from the review question?	High	Low	UNCLEAR
**Reference Standard [2 signaling question, 1 risk of bias, 1 applicability]**			
Is the reference standard likely to correctly classify the target condition?	YES	NO	UNCLEAR
Were the reference standard results interpreted without knowledge of the results of the index test?	YES	NO	UNCLEAR
Could the reference standard, its conduct, or its interpretation have introduced bias?	High	Low	UNCLEAR
Are there concerns that the target condition as defined by the reference standard does not match the review question?	High	Low	UNCLEAR
**Flow and Timing [4 signaling questions, 1 risk of bias]**			
Was there an appropriate interval between index test(s) and reference standard?	YES	NO	UNCLEAR
Did all patients receive a reference standard?	YES	NO	UNCLEAR
Did all patients receive the same reference standard?	YES	NO	UNCLEAR
Were all patients included in the analysis?	YES	NO	UNCLEAR
Could the patient have introduced bias?	High	Low	UNCLEAR

Note:

1. Applicability ratings "do not include signaling questions. Review authors record the information on which the judgment of applicability is made and then rate their concern that the study does not match the review question. Concerns about applicability are rated as "low", "high", or "unclear."" " The "unclear" category should be used only when insufficient data are reported."
2. Applicability in patient selection category can refer to concerns about: severity of illness, demographic characteristics, differential diagnoses, co-morbidities, setting of study, or previous testing.

3. If applicability concerns rated high or unclear, please describe reasons here:<br>Patient selection:<br>______________________________________<br>__________________________________<br>Index Test:<br>______________________________________<br>__________________________________<br>Reference Standard:<br>______________________________________<br>__________________________________	
Reliability (of index test to differentiate patients with from those without delirium) = observer variability.	Inter-rater:<br>Number of raters: _________<br>Number of replicate observations: _________<br>Method: intraclass correlation / Kappa statistic / other (_______________)<br>Result: _______ 95% CI _________
	Intra-rater:<br>Number of replicate observations: ______<br>Method: intraclass correlation / Kappa statistic/ other (_______________)<br>Result: _______95% CI _________
	Neither
Agreement (proportion of index test scores that are identical within or between raters)	Inter-rater:<br>Proportion of Agreement:<br>95% CI:<br>Other statistic: ____________________
	Intra-rater:<br>Proportion of agreement:<br>95% CI:<br>Other statistic: ____________________
	Neither

Name: ________________________________

Comments:
