# Supplementary material for: Diagnostic accuracy of delirium diagnosis in pediatric intensive care: a systematic review
Source: Crit Care. 2014 Sep 26;18(5):489. doi: 10.1186/s13054-014-0489-x (PMC4207322; doi:10.1186/s13054-014-0489-x)
Supplement: Additional file 3: — Supplemental statistical analysis and results section of the systematic review. This file provides details of planned analyses that were not performed due to lack of data in the included publications in the review, and details of the specific biases present in each included study. [file 13054_2014_489_MOESM3_ESM.pdf]

DIAGNOSTIC ACCURACY OF DELIRIUM DIAGNOSIS IN PEDIATRIC INTENSIVE CARE: A SYSTEMATIC REVIEW

ESM_3_Joffe:
Supplemental statistical analysis and results section of the systematic review..

Authors: Alia Daoud BSc[1], Jonathan P Duff MD[1,2], Ari R Joffe MD[1,2]

Affiliations: 1. University of Alberta, Faculty of Medicine and Dentistry; 2. University of Alberta, Department of Pediatrics and Stollery Children's Hospital.

Corresponding Author: Ari R Joffe MD; 4-546 Edmonton Clinic Health Academy; 11405 87 Avenue; Edmonton, Alberta, Canada; T6G 1C9. Phone: 780 2485435. Email: ari.joffe@albertahealthservices.ca Fax: 780 4073214.

Statistics: We planned to determine the following for each index-test: 1. Accuracy of index-tests, determined by sensitivity, specificity, positive and negative predictive value and likelihood ratios. If enough information is available, further summary statistics were planned. A summary ROC was to be constructed (an estimate of the underlying relationship between sensitivity and specificity for the test used across varying thresholds), with average sensitivity and specificity with 95% CIs (when included studies have used a common threshold of the same index test). Summary likelihood ratios were also to be calculated if possible from average sensitivity and specificity. If there were direct comparative analyses of index-tests, paired analyses displayed on ROC curves linking the sensitivity-specificity pairs from each study with a dashed line were to be made, and if possible the summary sensitivity and specificity compared by whether the 95% CI overlap. We also looked at the impact of inconclusive tests on the index-test accuracy [8]. 2. Reliability and Agreement of Index-Tests used as an indicator of the amount of measurement error inherent in the index-test score. Reliability measures the ability of the test to differentiate those with and without delirium. Agreement measures the degree to which test scores are identical. Both can be determined inter-rater, and intra-rater (test-retest), and depend on the context/setting of the test [i.e. are not fixed properties of the measurement tool]. Kappa statistics and intra-class correlation coefficients (for nominal or ordinal/continuous measurements respectively) for reliability, and proportion of agreement (including ranges or Bland Altman plots for continuous measurements) were to be described, with CIs. Kappa values 0.6-0.8 are considered sufficient for group level comparisons; however, for individual diagnosis and important decisions kappa should be at least 0.9. We examined for pre-defined risk factors for, incidence of (by the reference standard), and outcome of delirium reported in the identified diagnostic accuracy studies. Predefined risk factors for delirium were: sepsis, diagnostic category, co-morbidities, severity of illness scores, age, sex, and

medications, and using the individual study definitions and statistical testing employed. Pre-defined outcomes of delirium were: mortality, PICU and hospital length of stay, and long-term neuro-cognitive outcomes; if delirium as a predictor of any of these outcomes was examined in the study, this is described using the statistics employed in that study.

Results:

Table. Biases present in the 5 included studies of the accuracy of delirium screening tests in pediatric intensive care.

**Bias**	**Definition**	**Studies with the bias**
Spectrum	Included patients do not represent the intended spectrum of severity for the target condition or alternative conditions (eg. more advanced stages).	-
Selection	Eligible patients are not enrolled consecutively or randomly.	PAED (10/54 with missing data)
Index Test Information	The index test results are interpreted with knowledge of the reference test results, or with more clinical information than in practice.	PAED (imputed data for at least 16% of patients); CAP-D (R) (repeat assessments may have been after known diagnosis of delirium by psychiatry)
Misclassification	The reference standard does not correctly classify patients with the target condition.	-
Context	The prevalence of delirium is much different than expected in the PICU population.	p-CAM (only 6% ventilated); Clinical suspicion (prevalence

		only 4.6%)
Partial verification	A non-random set of patients does not undergo the reference standard test.	p-CAM (16/93 without paired data);<br>CAP-D (6/56 "incomplete data");<br>Clinical suspicion (reference standard only done on those with clinical suspicion)
Differential verification	A set of patients is verified with a different reference standard than another set, especially when this selection depends on the index test result (eg. index test influences decision to order reference test).	Clinical suspicion (clinical suspicion was what triggered decision for reference test)
Incorporation	The index test is incorporated in a composite reference standard.	Clinical suspicion (reference standard included discussion with the PICU bedside team, who had the clinical suspicion)
Disease progression	The patient's condition changes between administering the index test and the reference standard (eg. when the period of time between the index and	Clinical suspicion (multidisciplinary meeting may

	reference tests is too long; defined as >24hr apart, not on same day).	not have been on the same day as index test);<br>CAP-D (R) (unclear interval between index and reference test)
Reference test information	The reference standard is interpreted knowing the index test results.	Clinical suspicion (the psychiatrist knew the index test was positive, resulting in the referral for assessment)
Excluded data	Occurs when un-interpretable or intermediate test results and withdrawals are not included in the analysis.	PAED (for 10/154);<br>p-CAM (25/93 enrolled were excluded: 13 no paired assessment, 3 done over 3hr apart, 1 developmentally delayed, 7 with coma);<br>CAP-D (6/56 eligible patients with incomplete data)

Limited challenge	Patients with a specific condition known to adversely affect the way the index test works are excluded (eg. difficult to diagnose patients are excluded).	PAED (imputed for >16% of patients)

Overall, of the 12 types of bias, the number present in each study were: PAED, 4; p-CAM, 3; CAP-D, 2; CAP-D (R), 2; Clinical suspicion, 6.
